# Unlocking complex pathways in ventricular tachycardia: Ventricular abnormal activities detection mapping in a case of scar-related multiple ventricular tachycardias

**DOI:** 10.1016/j.hrcr.2023.12.002

**Published:** 2023-12-07

**Authors:** Hiroyuki Kono, Kenichi Hiroshima, Michio Nagashima, Masato Fukunaga, Kengo Korai, Kenji Ando

**Affiliations:** Department of Cardiology, Kokura Memorial Hospital, Kitakyushu, Japan

**Keywords:** Ventricular tachycardia, Scar-related, Radiofrequency ablation, Functional map, Ischemic cardiomyopathy, Implantable cardioverter-defibrillator

**Key Teaching Points**

• Ventricular abnormal activities detection mapping using the “early meets late” function in the CARTO system effectively identifies slow conduction patterns such as delayed potential and local abnormal ventricular activities without oversight.
• By performing a pace map in areas where abnormal potentials correlate with slow conduction and ensuring it aligns with the ventricular tachycardia (VT) waveform, we can effectively estimate the location of the critical isthmus.
• In cases with a high prevalence of slow conduction, the lower threshold of “early meets late” can be elevated to enhance the detection of abnormal potentials while retaining specificity. This approach is especially useful in situations where various types of VT are induced, or when the critical isthmus exists on both the endocardial and epicardial side.

## Introduction

Ventricular tachycardia (VT) represents a critical and often fatal cardiac disease. Although implantable cardioverter-defibrillator (ICD) implantation has emerged as an effective treatment modality, it falls short in preventing the onset of the disease itself.^1^ Recent studies have demonstrated the efficacy of ablation in preventing the recurrence of VT.^2^ If mapping during VT is achievable, or if good pace mapping is obtained, ablation may be carried out by pinpointing the critical isthmus.^3^^,^^4^ However, this process becomes complex in VT cases accompanied by hemodynamic instability or poor inducibility.

Substrate mapping, accomplished through the ablation of the abnormal local activities such as delayed potential (DP) and local abnormal ventricular activities (LAVAs), has been substantiated to enhance results.^5^^,^^6^ Additionally, functional mapping has been reported to be advantageous for circuit estimation. Isochronal late activation mapping (ILAM) is able to recognize the deceleration zone during sinus rhythm.^7^ Moreover, rotational activation patterns (RAP) are known to exhibit a relationship with the critical isthmus.^8^

However, the identification of slow conduction becomes intricate when multiple VTs are present, when determining the exit/entrance site of the VT is problematic, or when endocardial (Endo) /epicardial (Epi) elements are included in the circuit. Therefore, the mapping strategy aims to discern the slow conductions that could constitute the critical isthmus, striving for precision and simplicity.

### Ventricular abnormal activities detection map: An overview

Using the “early meets late” function in the CARTO system (Biosense Webster, Diamond Bar, CA), a ventricular activation abnormalities detection (VAAD) map is constructed during sinus rhythm or high right atrial pacing. This approach is predicated on the total activation time (TAT) within the observed window, the variance in activation time between the 2 acquired potentials, and the fraction of the TAT conforming to a lower threshold setting.

To detect abnormal local potentials such as DP and LAVAs, the lower threshold is strategically set within a range of 10%–30%, allowing for a comparative analysis of the outcomes. In instances where there is a high prevalence of slow conduction, the specificity of detection is enhanced by elevating the lower threshold. On the other hand, when slow conduction points are infrequent, the sensitivity of detection is improved by decreasing the lower threshold. The optimal value for the lower threshold is determined by the abnormal potentials of the critical isthmus. This comprehensive technique is referred to as the VAAD map, providing a robust substrate mapping strategy for understanding and treating complex VT.

## Case report

A 66-year-old male patient, who had a myocardial infarction in the left anterior descending branch (#6) 38 years prior, underwent an ICD implantation owing to sustained VT episodes 15 years ago. He had undergone catheter ablation for scar-related VT in 4 sessions, with all procedures exclusively targeting the Endo side.

During this admission, he presented with an electrical storm, accompanied by recurrent VT episodes. His medications included warfarin, sotalol, bisoprolol, enalapril, and spironolactone. Blood tests revealed a creatinine level of 0.78 mg/dL, a hemoglobin level of 13.2 g/dL, and a brain natriuretic peptide level of 60.3 pg/mL. The chest radiograph indicated an implanted ICD in the right anterior thoracic region and a cardiothoracic ratio of 55%. A 12-lead electrocardiogram exhibited an atrial pacing waveform, while echocardiographic findings showed left ventricular end-diastolic diameter of 66 mm, end-systolic diameter of 53 mm, left ventricular ejection fraction of 33%, and no significant valvular disease. Coronary computed tomography revealed a truncated left anterior descending branch without other stenoses. The 12-lead electrocardiogram during the VT episode (clinical VT) displayed a downward axis with a right bundle branch block pattern. The peak deflection index measured 0.74, indicating the involvement of the Epi side in the VT circuits.

**Figure 1:**
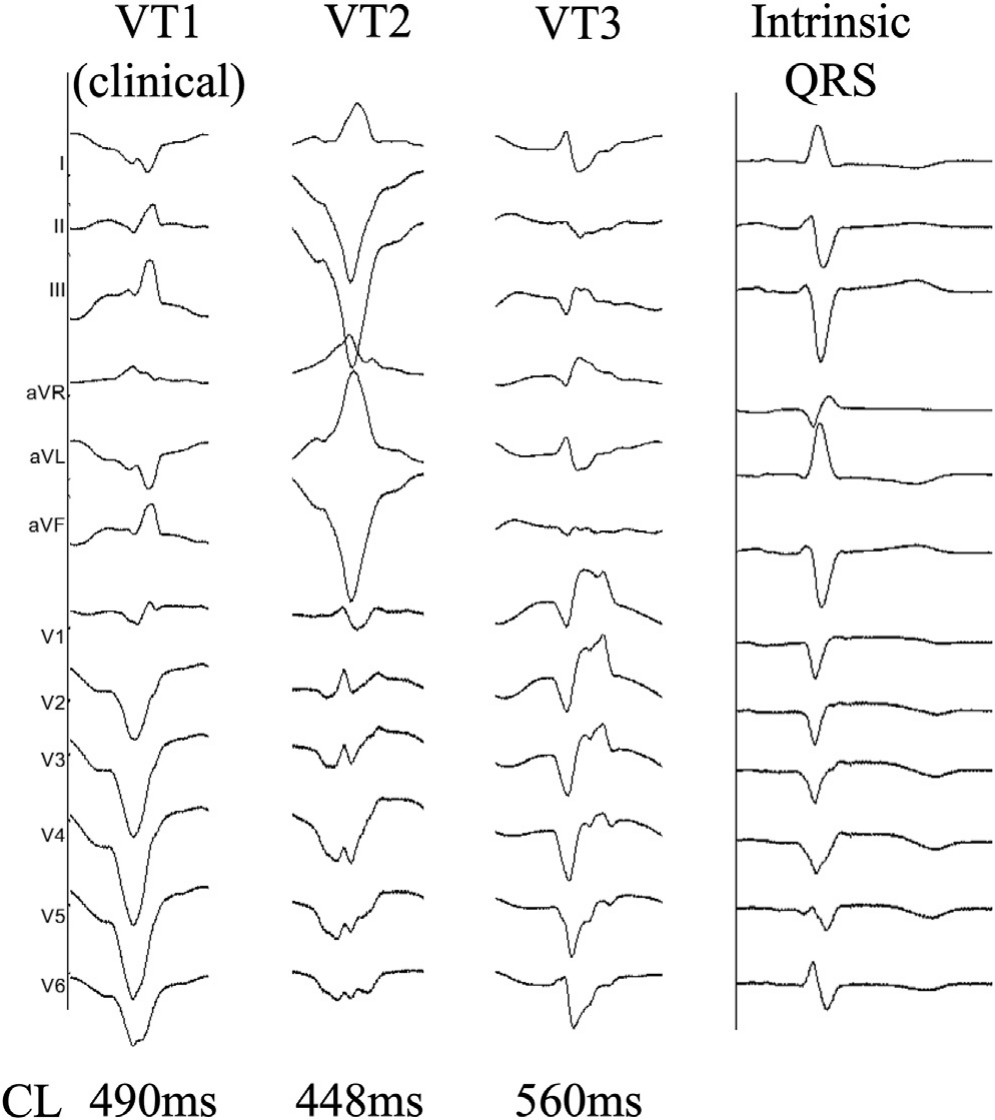
Three types of induced ventricular tachycardia (VT) and intrinsic QRS waveform during the atrium pacing. The cycle length (CL) is shown below. VT1 was identical to clinical VT.

During the ablation procedure, we induced 3 distinct VT patterns (Figure 1). VT1 was identical to the clinical VT (cycle length [CL] 490 ms); VT2 exhibited an upper axis, negative concordant pattern (CL 448 ms); VT3 presented a slightly upper axis with a right bundle branch block pattern (CL 560 ms). VT1 demonstrated hemodynamic instability, while VT3 was poorly induced, allowing mapping only during VT2.

**Figure 2:**
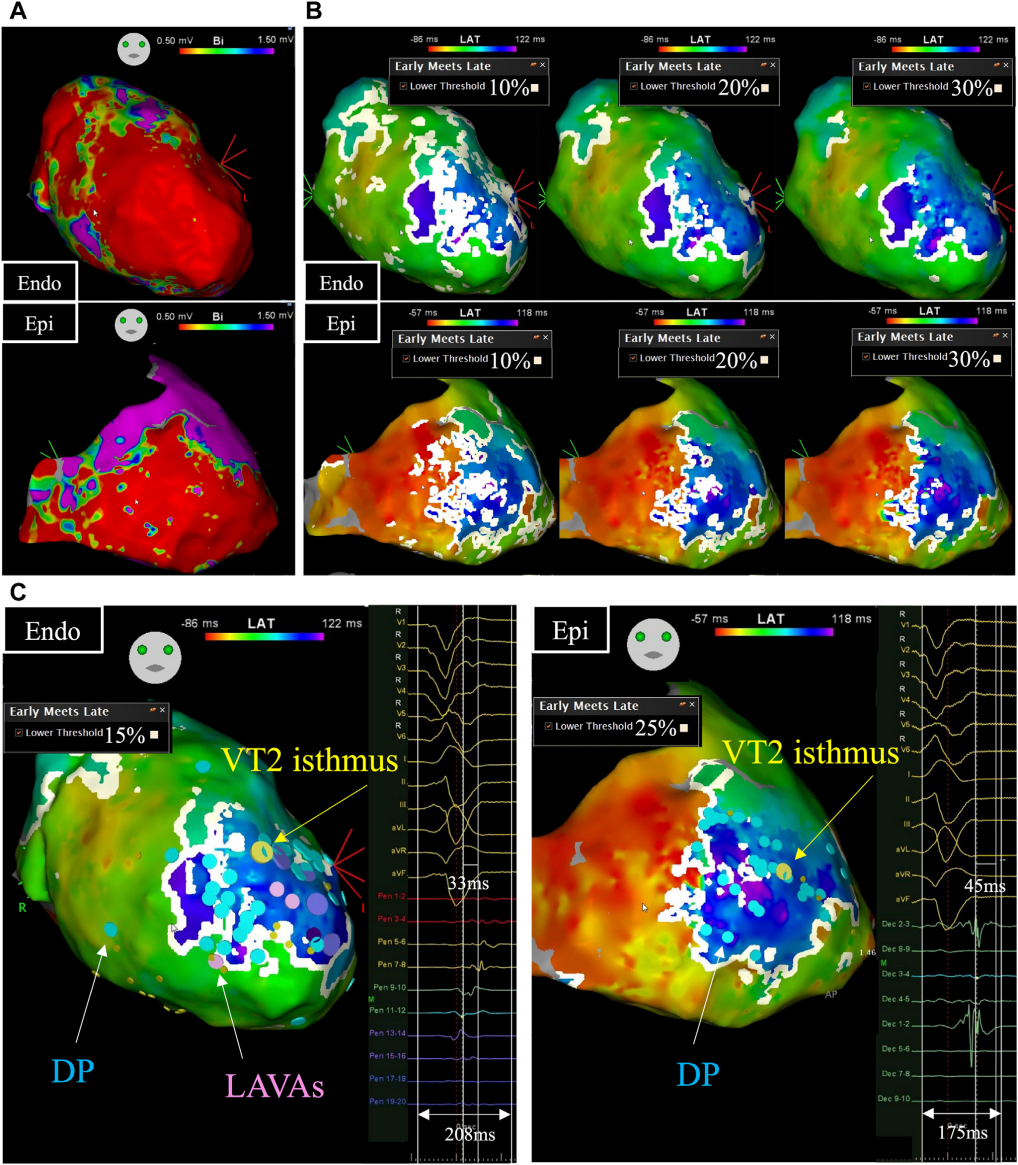
Voltage map and ventricular abnormal activities detection (VAAD) map adjusting the lower threshold of “early meets late” function. **A:** Voltage map shows an extensive low-voltage zone in both the endocardial (Endo) and epicardial (Epi) side, reflecting the myocardial infarction in the left anterior descending branch region. **B:** VAAD map adjusting the lower threshold from 10% to 30%. The left image with a lower threshold of 10% shows subtle slow conduction over a large area within the low-voltage zone. The right image with a lower threshold of 30% depicts pronounced slow conduction, which is less prevalent. **C:** We adopted the VAAD map to detect abnormal activities, with a lower threshold of 15% set for the Endo side and 25% for the Epi side. These values were established by assessing the critical isthmus sites. On the Endo side, the variance between Pentaray 7-8 and Pentaray 13-14 was 33 ms, compared to a total activation time (TAT) of 208 ms (15.9%). On the Epi side, the variance between Decanav 2-3 and Decanav 3-4 was 45 ms relative to a TAT of 175 ms (25.7%). Delayed potential (DP), marked by a blue tag; local abnormal ventricular activities (LAVAs), marked by a purple tag; and VT2 isthmus, marked by a yellow tag, were all adjacent to the highlighted line on the VAAD map.

**Figure 3:**
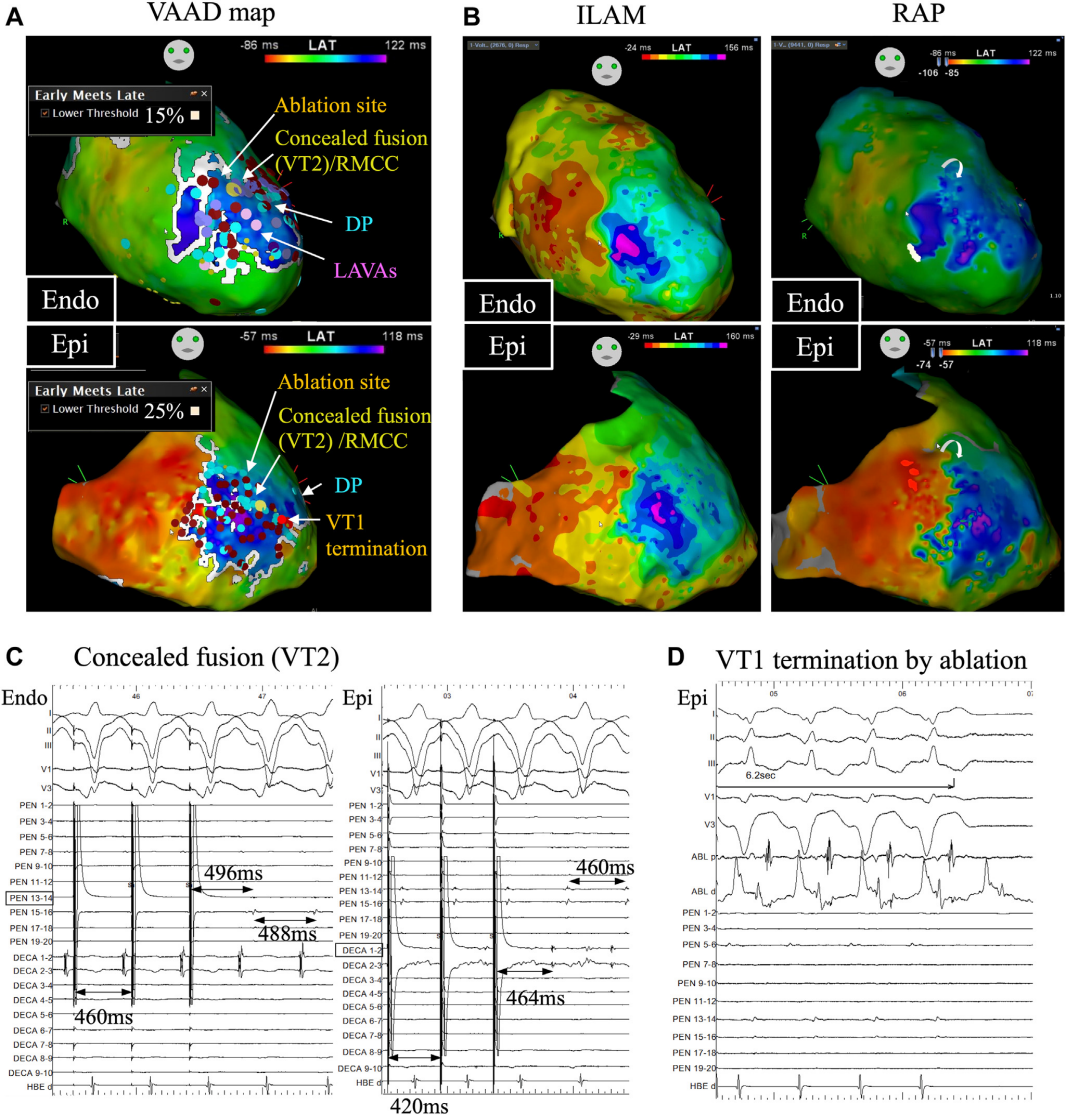
The relationship between ventricular abnormal activities detection (VAAD) map and abnormal potentials in comparison with the functional maps. **A:** The VAAD map integrated with the ablation lesion set. Delayed potential (DP) is denoted by a blue tag, local abnormal ventricular activities (LAVAs) by a purple tag, and the ablation points by a dull red tag. A ripple mapping conduction channel (RMCC) between the endocardial (Endo) and epicardial (Epi) side was identified at the location marked by the yellow tag. At this site, an optimal pace map was achieved, and concealed fusion was observed during entrainment pacing for VT2. The site of VT1 termination by ablation is marked by the red tag. **B:** Isochronal late activation mapping (ILAM) and rotational activation pattern (RAP). ILAM shows 2 deceleration zones at the apex. RAP exists on the upper side of the apex. **C:** Entrainment pacing during VT2 shows concealed fusion with the postpacing interval nearly equal to the total cycle length on both the Endo and Epi sides. **D:** Mid-diastolic potential during VT1 was observed at the proximal electrode of the ablation catheter. VT1 was terminated after 6.2 seconds of ablation.

The left ventricular Endo side was mapped using a multielectrode catheter (Pentaray®, Biosense Webster, Diamond Bar, CA) and Epi side using a multielectrode catheter (DECANAV®, Biosense Webster, Diamond Bar, CA) during atrial pacing by ICD. There was an extensive low-voltage zone at the anterior wall and apex, which posed challenges in estimating abnormal potentials and identifying the critical isthmus (Figure 2A). We conducted a VAAD map, adjusting the lower threshold of “early meets late” from 10% to 30% (Figure 2B). Both the Endo and Epi showed a U-shaped highlighted line correlating with slow conduction. A reduction in the lower threshold might detect subtle slow conduction, albeit sacrificing specificity. In contrast, a heightened lower threshold, while potentially less sensitive, can discern more definite slow conduction. We estimated the critical isthmus of VT2 on both the Endo and Epi sides using pace mapping and entrainment pacing, as detailed later. The variance in the local activation time of DP at this site was 33 ms, compared to a TAT of 208 ms (15.9%) on the Endo side. On the Epi side, the variance was 45 ms relative to a TAT of 175 ms (25.7%). As a result, we set a 15% lower threshold for the Endo side and a 25% lower threshold for the Epi side (Figure 2C). DP and LAVAs were tagged manually, which had a good correlation with the highlighted line in the VAAD map. A pace map performed at the site of abnormal local potentials exhibited the same waveform as VT1 on the Epi side. Stimulation to QRS (S-QRS) was 120 ms (S-QRS/CL: 24%), which meant exit site of VT1. Similarly, good pace maps were obtained for VT2 with S-QRS measurements of 96 ms on the Endo side and 120 ms on the Epi side. At this site, ripple map conduction channels were observed between the Endo and the Epi, as indicated by the yellow tag. During sustained VT2, we conducted a propagation map only on the Epi side, leading to the identification of the critical isthmus in a figure-8 configuration. At the site of the good pace map for VT2, we achieved entrainment pacing and observed concealed fusion with the postpacing interval nearly equal to the total cycle length (Figure 3C). At the site of the good pace map for VT1, mid-diastolic potential during VT1 was observed. VT1, being unmappable owing to hemodynamic instability, was terminated after 6.2 seconds of ablation (Figure 3D). Following DP and LAVA ablation, VT was no longer inducible. The location of the ablation lesion is indicated by the dull red tag in Figure 3A.

## Discussion

The current case involved scar-related VT with multiple VTs including both the Endo and Epi sides. There are several methodologies for detecting critical isthmus and targeting the noninducibility of VT. For substrate mapping, the abolition of DP and LAVA has proven to be effective and feasible.^5^^,^^6^ However, maintaining reproducibility remains challenging. In this case, the critical isthmus for both VT1 and VT2 was identified by pace map in the slow conduction region where the VAAD map highlighted DP. The critical isthmus was strategically positioned proximal to the highlighted zone on the VAAD map, suggesting the efficacy of this mapping modality in the investigative process.

In 95% of cases, the deceleration zone identified by ILAM correlated well with the critical isthmus.^7^ RAP demonstrated a sensitivity of 70% and specificity of 89% in predicting elements within the critical isthmus for VT.^8^ We employed both ILAM and RAP for VT analysis (Figure 3B). Although ILAM identified 2 deceleration zones, they did not correspond with the critical isthmus site. Notably, while ILAM is primarily used with the Ensite system (Abbott Medical, Abbott Park, IL), its real-time application in the CARTO system remains challenging. Similarly, RAP did not align with the critical isthmus. Both ILAM and RAP typically exhibit 1 or 2 points within the slow conduction zone, adhering to an all-or-nothing manner. These modalities do not provide the continuous detail required to discern subtle slow conduction patterns associated with the critical isthmus in complicated cases. Further, the conducting channel through Endo/Epi can be identified using ripple mapping, aiding in detecting the intramural circuit.^9^

In the Ensite system, the fractionation map has been reported to be beneficial for substrate modification and in reducing procedural time.^10^ Combination of this with conduction velocity maps and ILAM has been shown to enhance substrate modification and more accurately identify the critical isthmus.^11^ In the Rhythmia system (Boston Scientific, Marlborough, MA), the presence of prolonged electrogram durations has been identified as advantageous for substrate modification.^12^ Both systems offer the capability to adjust the threshold for fractionation signals, aiding in substrate modification. In contrast, the “early meets late” function, exclusive to the CARTO system, enables the elucidation of local conduction delays as manifested in LAVAs, DP, and critical isthmuses by highlighting differences between local potentials.

The sensitivity and specificity of detecting DP, LAVAs, and critical isthmus by the VAAD map could be modulated by adjusting the lower threshold of “early meets late” function, allowing a progressive and relative accentuation of abnormal local potentials. The ability to adjust the VAAD map’s detection sensitivity makes it especially useful in situations where various types of VT are induced, or when the critical isthmus exists on both the Endo and Epi side.

## Conclusion

The success of this complex case underscores the pivotal role of cutting-edge VAAD mapping strategies in managing intricate VT. By adeptly employing “early meets late” function to identify abnormal potentials, we were able to discern multiple VT circuits and DP through both Endo and Epi layers without oversight. The insights derived from this case may fuel further innovations in VT mapping and treatment, promoting individualized and efficacious therapeutic approaches for multiple complex VT cases.
